# Maintain Genomic Stability: Multitask of DNA Replication Proteins

**DOI:** 10.4172/2329-8936.1000e108

**Published:** 2015-01-31

**Authors:** Jing Hao, Wenge Zhu

**Affiliations:** Department of Biochemistry and Molecular Medicine, The George Washington University Medical School, 2300 Eye Street N.W., Washington, DC 20037, USA

## Editorial

Maintenance of genomic stability is critical for living organisms because it is crucial for cell survival and development, and it prevents the development of deleterious mutations. Overriding this control will cause genomic instability, a hallmark of cancer.

**Figure 1:**
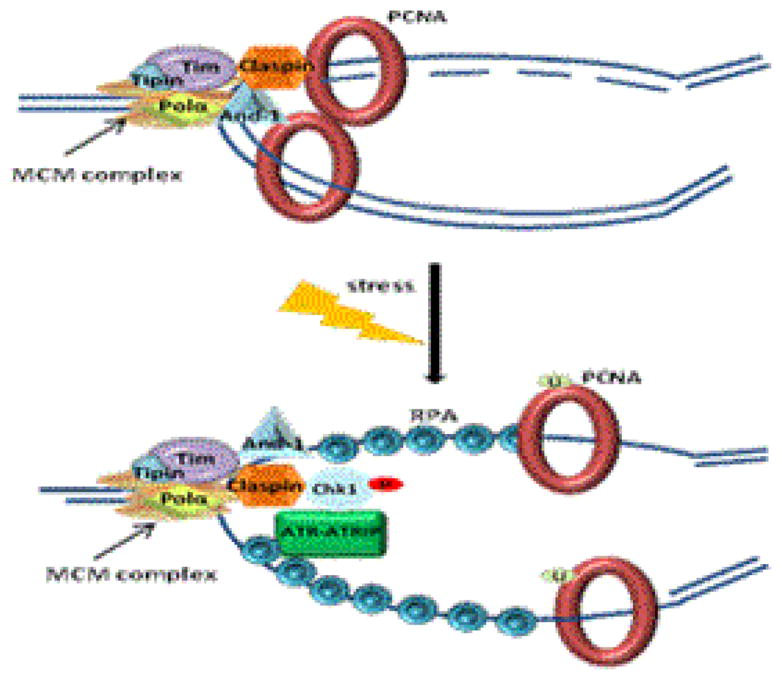
Cells with DNA damage such as replication stress, replication forks are stalled

The genome is highly vulnerable to damage, especially during DNA replication because chromosome is decondensed and the replication forks are extremely sensitive to DNA damage agents. The eukaryotic replisome, which consists of a large number of replication fork-associated proteins, is essential for the elongation of replication forks during DNA replication. This complex contains DNA polymerases, MCM helicase, single stranded DNA (ssDNA) binding protein RPA, sliding clamp PCNA, Tipin, Timeless, Claspin, And-1, etc. In cells with DNA damage such as replication stress, replication forks are stalled Figure 1. At stalled replication forks, some of replisome components switch their role from facilitating DNA synthesis to inducing activation of DNA replication checkpoint, a signaling transduction pathway that is critical to maintain fork stability and triggers cell cycle arrest.

Specifically, DNA lesions induced by replication stress lead to replication fork stalling, and at stalled replication forks ssDNA and primer-template junctions are formed [1]. ssDNA is generated when DNA helicase and DNA polymerase activities become uncoupled from one another due to either physical obstructions or nucleotide deficiencies that block DNA polymerase progression [2]. The replication and checkpoint protein TopBP1 cooperates with the BACH1/FANCJ helicase to promote loading of RPA onto ssDNA at the stalled replication forks [3]. The ATR kinase is then recruited to the stalled replication forks through a specific interaction between RPA and ATR-interacting proten ATRIP [4]. The ATR is activated by a mechanism involving Rad9/Hus1/Rad1 (9-1-1) clamp and TopBP1 [5,6]. Ultimately, coordinated actions of several replisome proteins, including Claspin, Timeless, and Tipin, bring Chk1 to the stalled forks to be phosphorylated by ATR [7,12].

Timeless and Tipin proteins form heterodimers and interact with MCM helicase [7,13]. Depletion of either one compromises S phase checkpoint activation [7,14]. Intriguingly, Timeless-Tipin complex prevents ssDNA accumulation at unperturbed replication forks and facilitates activation of ATR-Chk1 pathway in cells experiencing replication stress [15]. Claspin binds to DNA at the replication origins and is important for DNA synthesis in unperturbed cells [16]. Importantly, Claspin is a Chk1-interacting protein and Claspin-Chk1 interaction is critical for Chk1 phosphorylation under conditions that causes replication stress [11]. Timely degradation of Claspin is needed to release the cells from checkpoint arrest, enabling them to resume DNA replication [17,18]. In addition, Claspin, Timless and Chk1 are also known to regulate PCNA ubiquitination to tolerate DNA damage and avoid catastrophic replication fork collapse [19,20].

Replication checkpoint can prevent synthesis of damaged DNA and maintain the stability of stalled replication forks. For example, nuclease Exo1 and helicase SMARCAL1 are inhibited following activation of the replication checkpoint activation as a way to stabilize replication forks [21,22]. In cells with replication stress, checkpoint prevents DNA synthesis by inhibiting late origin firing [23,24]. As a key regulator of checkpoint activation, ATR has long been established to maintain replication fork stability and to prevent replication origin firing during replication stress. Recent study showed that ATR prohibits replication catastrophe by preventing global exhaustion of RPA [25]. However, it should be noted that RPA exhaustion in the presence of ATR can still lead to irreversible breakage of replication forks [25].

Replication proteins play multiple roles in cells. In addition to their role in DNA synthesis, replisome proteins are essential for DNA replication checkpoint activation and maintenance of fork stability. Although it is clear that replisome proteins are critical for replication checkpoint activation, it remains largely unknown that how replisome components coordinate with checkpoint proteins during checkpoint activation. It also remains to be determined how checkpoint pathway targets replisome proteins to maintain fork stability in cells with replication stress. Addressing these questions will significantly help us to understand the role of replication proteins in the regulation of genomic stability.
